# A Personalized Approach to Biological Therapy Using Prediction of Clinical Response Based on MRP8/14 Serum Complex Levels in Rheumatoid Arthritis Patients

**DOI:** 10.1371/journal.pone.0152362

**Published:** 2016-03-30

**Authors:** S. C. Nair, P. M. J. Welsing, I. Y. K. Choi, J. Roth, D. Holzinger, J. W. J. Bijlsma, J. M. van Laar, D. M. Gerlag, F. P. J. G. Lafeber, P. P. Tak

**Affiliations:** 1 Department of Rheumatology and Clinical Immunology, University Medical Center, Utrecht, The Netherlands; 2 Division of Clinical Immunology and Rheumatology, Academic Medical Center/University of Amsterdam, Amsterdam, the Netherlands; 3 Institute of Immunology, University Hospital Muenster, Muenster, Germany; 4 Department of Pediatric Rheumatology and Immunology, University Children's Hospital Muenster, Muenster, Germany; Nippon Medical School Graduate School of Medicine, JAPAN

## Abstract

**Objectives:**

Measurement of MRP8/14 serum levels has shown potential in predicting clinical response to different biological agents in rheumatoid arthritis (RA). We aimed to develop a treatment algorithm based on a prediction score using MRP8/14 measurements and clinical parameters predictive for response to different biological agents.

**Methods:**

Baseline serum levels of MRP8/14 were measured in 170 patients starting treatment with infliximab, adalimumab or rituximab. We used logistic regression analysis to develop a predictive score for clinical response at 16 weeks. MRP8/14 levels along with clinical variables at baseline were investigated. We also investigated how the predictive effect of MRP8/14 was modified by drug type. A treatment algorithm was developed based on categorizing the expected response per drug type as high, intermediate or low for each patient and optimal treatment was defined. Finally, we present the utility of using this treatment algorithm in clinical practice.

**Results:**

The probability of response increased with higher baseline MRP8/14 complex levels (OR = 1.39), differentially between the TNF-blockers and rituximab (OR of interaction term = 0.78), and also increased with higher DAS28 at baseline (OR = 1.28). Rheumatoid factor positivity, functional disability (a higher HAQ), and previous use of a TNF-inhibitor decreased the probability of response. Based on the treatment algorithm 80 patients would have been recommended for anti-TNF treatment, 8 for rituximab, 13 for another biological treatment (other than TNFi or rituximab) and for 69 no recommendation was made. The predicted response rates matched the observed response in the cohort well. On group level the predicted response based on the algorithm resulted in a modest 10% higher response rate in our cohort with much higher differences in response probability in individual patients treated contrary to treatment recommendation.

**Conclusions:**

Prediction of response using MRP8/14 levels along with clinical predictors has potential in personalizing treatment for RA patients starting biological anti-rheumatic treatment, and might increase cost-effectiveness.

## Introduction

Biological therapies have become commonly available for the treatment of rheumatoid arthritis (RA) over the past decades.[1] Biologics are considered in RA patients with active disease in spite of treatment with synthetic disease-modifying antirheumatic drugs (DMARDs), including methotrexate (MTX).[2] Tumor necrosis factor (TNF) inhibitors,[3–7] rituximab (a B cell depleting anti-CD20 antibody),[5] abatacept (a selective T cell co-stimulation modulator),[8] and tocilizumab (an anti-interleukin (IL) 6 receptor antibody),[9] have been approved for the treatment of RA. In clinical practice these biologicals are sometimes used in a ‘trial-and-error’ fashion, the order mainly based on payers’ or regulatory restrictions. In most cases a TNF-inhibitor is started, followed by either another TNF-inhibitor or a biological with another mechanism of action when insufficient treatment response is observed or when treatment response is lost over time. On the group level all biological therapies exert more or less the same clinical effect with about two thirds of the patients responding (moderate to good) to treatment as determined using the European League Against Rheumatism (EULAR) or American College of Rheumatology (ACR) response criteria.[10] However, the individual patients who respond to one mechanism of action are not necessarily the same as those responding to another.[11] Stratifying patients in order to increase the chance of a robust treatment effect, will lower the chance of side effects of ineffective treatment and increase cost-effectiveness which is specifically relevant for these relatively expensive drugs. It may also provide insights into different mechanisms of disease in these patient subgroups.[11, 12] Specific biomarkers related to the disease process might be helpful in the context of individualized health care.

Tools which can be used in daily practice to predict response to biological drugs and guide the choice of treatment are relatively scarce. Although many studies have explored predictive factors for response to biological therapies, only few have been confirmed.[11] Conceivably, prediction models may be improved by combining measurement of biomarkers with clinical parameters. Recent work has shown that serum concentrations of myeloid related protein 8 and 14 (MRP8/14) protein complex are a promising biomarker to predict response to biological therapy in active RA patients at baseline and could be used to monitor response to treatment across different mechanisms of action.[13, 14] MRP8/14 protein complex significantly contributes to joint inflammation and leucocyte infiltration[15] and has also been proposed as biomarker to monitor disease activity in many other inflammatory diseases and is able to detect subclinical inflammation.[16–18] It has been suggested that MRP8/14 levels may be superior to CRP levels for monitoring ultrasound-determined synovial inflammation in RA patients. [19] In the current study we investigated the predictive value of MRP8/14 serum levels for clinical response to treatment when combined with clinical parameters like rheumatoid factor and baseline disease activity. Moreover, using the resulting predictive score we developed a treatment algorithm for individual patients with active RA for whom biological treatment is considered. This treatment algorithm could facilitate improved treatment decision with biologicals in RA patients.

## Methods

### Description of the data used

We used a previously published dataset to determine the association between serum levels of MRP8/14 at baseline and the clinical response to treatment with infliximab, adalimumab and, rituximab.[13] 170 patients were included in this study of which eighty-six patients were treated with adalimumab, 60 with infliximab, and 24 with rituximab. MRP8/14 serum complexes were measured at baseline after initiation of treatment. Clinical response was determined according to the EULAR response criteria at baseline and at 16 weeks (24 weeks for rituximab). Furthermore, we evaluated the 28 joint count disease activity score (DAS28), C-reactive protein (CRP) levels, immunoglobulin M rheumatoid factor (IgM RF), anti–citrullinated protein antibodies (ACPA), health assessment questionnaire disability index (HAQ), age, gender, and treatment history with biologicals at baseline as predictive factors.

### Development of a predictive score for response on adalimumab, infliximab, and rituximab

Although it has previously been found that MRP8/14 complexes are associated with clinical response to adalimumab, infliximab and rituximab separately,[13] it has as yet not been investigated if and how MRP8/14 levels predict the clinical response differently between these drugs or drug classes. This information is crucial in clinical practice for making a choice between these drugs or drug classes when biological treatment is indicated.

To test whether there are differences in prediction of response by MRP8/14 levels between different biologicals, we calculated the discriminative ability of MRP8/14 complexes for EULAR moderate or good response (defined as EULAR moderate or good response) to treatment at 4–5 months. This was done separately for adalimumab, infliximab, and rituximab using the area under the receiver operating curve (ROC), as described before [13]. Differences between AUCs were tested for statistical significance. Next, we investigated the predictive effect of MRP8/14 levels for response as well as the modification of this predictive effect by treatment using logistic regression.

Based on the results of the above analysis we either grouped the drugs (per class: TNF-inhibitors or anti-B cell treatment) or used them separately in the logistic regression analysis to develop the predictive score for EULAR response. We built the predictive model using logistic regression in a stepwise manner with in the first step the variables age, gender, IgM RF, ACPA, DAS28, HAQ, MRP4/18 complex level, previous use of TNF inhibitors and the type of drug in the model. Next, we removed variables one by one, based on p value and regression coefficient to arrive at a final model. We also investigated modification of the predictive effect of MRP8/14 serum levels by the other predictors in the final model. A liberal p value of <0.3 was considered for statistical significance for this predictive analysis. To correct the regression coefficients for over fitting and internally validate the model, we used bootstrapping and the bootstrapped version of the model was used to compare predicted and observed response rates graphically to assess model fit.

### Development of the algorithm for personalized treatment

The validated predictive model (logistic regression function) was used to calculate predicted probabilities (i.e. chances) for EULAR response for each patient for treatment with each biological (class), by filling in the specific drug type in the predictive model and using the following formula: 1/ 1+e^-((5,845*Drugtype)-(0,633*RF)-(0,598*HAQBL)+(0,36*BLM)-(0,271*(drugtype*BLM))-(5,419)-(0,22*DASBL))^. When predicted probabilities for rituximab are calculated drug type = 0 and when predicted probabilities for TNF blockers are calculated drug type = 1. Using these predicted scores, each patient was classified as having a high, intermediate or low predicted probability of EULAR response for each biological. We considered a predicted probability for response below 0.5 to be a low chance and a predicted probability above 0.9 as a high chance, with probabilities in between considered as an intermediate chance of response. This classification was based on the average response in the total population being 74.1% and clear deviations (of about 25%) from this average response. Based on the expected (i.e. predicted) response categories for each treatment modality as mentioned above, we defined the optimal treatment/treatments, providing the treatment advice in each patient.

The added value of using the algorithm was calculated by comparing the average (mean) predicted response in the cohort based with recommended treatment with the observed average response in the cohort and with the predicted response with treatment contrary to the treatment advice.

All statistical analyses were performed in SPSS version 20 and SAS version 9.2

## Results

Table 1 shows the baseline characteristics of the study population, total and separate per drug that the patients received. Patients treated with rituximab were as expected more often IgM RF positive and had higher levels of HAQ and DAS28 at baseline compared to the other two cohorts. Previous use of TNF inhibitors was also higher in the rituximab group.

**Table 1:** Baseline patient characteristics and response at 16 weeks and 24 weeks.

Item		Total	Infliximab	adalimumab	Rituximab	p-value
	N	n = 170	n = 60	n = 86	n = 24	
**Female, n (%)**	170	129 (75.9)	43 (71.7)	67 (77.9)	19 (79.2)	**0.632**
**IgM RF positive, n (%)**	169	112 (66.3)	44 (74.6)	48 (55.9)	20 (83.3)	**0.010**
**ACPA positive, n (%)**	170	123 (72.4)	42 (70.0)	59 (68.6)	22 (91.7)	**0.073**
**Age, mean (sd)**	170	53.6 (12.7)	55.0 (12.8)	52.4 (12.6)	54.2 (12.5)	**0.440**
**ESR, median (Q1-Q3)**	170	22.5 (11.8–37.3)	25.5 (14.0–40.3)	19.5 (11.0–32.5)	34.0 (18.5–36.5)	**0.091**
**CRP, median(Q1-Q3)**	169	9.0 (3.7–22.0)	10.0 (4.0–23.0)	6.9 (2.4–15.7)	18.8 (7.9–40.0)	**0.003**
**SJC28, mean (sd)**	170	8.9 (5.3)	11.4 (5.5)	6.7 (4.0)	10.3 (6.1)	**0.000**
**TJC28, mean (sd)**	170	12.2 (7.0)	11.5 (6.9)	11.5 (6.9)	16.6 (6.6)	**0.004**
**VASGH, mean (sd)**	170	60.9 (21.0)	59.4 (21.3)	58.9 (20.6)	71.7 (19.2)	**0.023**
**DAS28, mean (sd)**	170	5.6 (1.1)	5.7 (1.1)	5.4 (1.0)	6.4 (1.1)	**0.000**
**HAQ, mean (sd)**	169	1.40 (0.7)	1.4 (0.7)	1.3 (0.7)	1.8 (0.7)	**0.011**
**MRP8/14, median (Q1-Q3)**	166	1315.0 (800.0–2457.5)	2027.5 (1220.0–3522.5)	995.0 (627.5–1482.5)	1665.0 (1035.0–2825.3)	**0.000**
**Previous TNF use, n (%)**	170	137 (80.6)	0 (0)	13 (15)	20 (83.3)	**0.000**
Response at 16 weeks and rituximab 24 weeks						
**DAS28, mean (sd)**		3.8 (1.4)	3.8 (1.5)	3.6 (1.3)	4.8 (1.4)	**0.001**
**ΔDAS28, mean (sd)**	167	1.8 (1.3)	1.9 (1.2)	1.8 (1.3)	1.6 (1.1)	**0.576**
**EULAR resp, n (%)**	166					
**Good**		64 (37.6)	25 (41.7)	36 (41.9)	3 (12.5)	**0.099**
**Moderate**		62 (36.5)	20 (33.3)	29 (33.7)	13 (54.2)	
**No response**		40 (23.5)	12 (20.0)	21 (24.4)	7 (29.2)	

IgMRF: immunoglobulin M rheumatoid factor; ACPA: anti–citrullinated protein antibodies; ESR: erythrocyte sedimentation rate; CRP: C-reactive protein; SJC28: 28 swollen joint count; TJC28:28 tender joint count; VASGH: visual analogous scale general health; DAS28: 28 joint count disease activity score; HAQ: health assessment questionnaire; MRP8/14: myeloid protein complex; EULAR: the European league against rheumatism response criteria

### Development of a predictive score for response on adalimumab, infliximab, and rituximab

The discriminative ability of MRP8/14 baseline serum levels for EULAR response was comparable for adalimumab and infliximab [13] with AUC of about 0.69 and 0.79 respectively, and was statistically significantly different for rituximab with an AUC of 0.98. Regarding the different predictive effect of MRP8/14 on outcome between drugs, we found this to be comparable for infliximab and adalimumab (combined regression coefficient of 0.085) and notably different for rituximab (regression coefficient 1.732) although this difference was not statistically significant when corrected for baseline DAS28 and IgM RF (p = 0.4). Based on this analysis we decided to combine the TNF-inhibiting drugs in one class and keep the interaction-term for a different predictive effect of MRP8/14 between drug classes in the model.

The logistic regression analysis resulted in a final model after internal validation using bootstrapping as shown in Table 2. The probability of EULAR response increased with higher MRP8/14 serum levels, and higher DAS28 at baseline. Positivity for IgM RF, higher HAQ and previous use of a TNF-inhibitor decreased the probability of response. Treatment with TNF-inhibitors increased the probability of response (EULAR) i.e. the chance of responding was higher in this group of patients. The positive predictive effect of the MRP8/14 complex level was less with this drug class. (**Table 2**)

**Table 2:** Logistic regression model after internal validation.

Predictors	Β	p-value
Constant	-5.32	0.07
IgM RF positivity	-0.47	0.28
DAS28 baseline	0.25	0.29
HAQ baseline	-0.63	0.08
TNF drug type	4.70	0.07
MRP8/14	0.33	0.03
MRP8/14* TNF drug type#	-0.5	0.11
Previous TNF used	-1.18	0.06

#MRP8/14*TNF drug type: this indicates the level of modification of the predictive effect of MRP8/14 when the drug is of the TNF-inhibitor type instead of rituximab.

IgM RF: immunoglobulin M rheumatoid arthritis, DAS28: 28 joint count disease activity score, HAQ: health assessment questionnaire, TNF: tumour necrosis factor, MRP814: baseline MRP8/14 complex

β: regression coefficient of logistic regression model

### Development of the algorithm for personalized treatment

**Fig 1:**
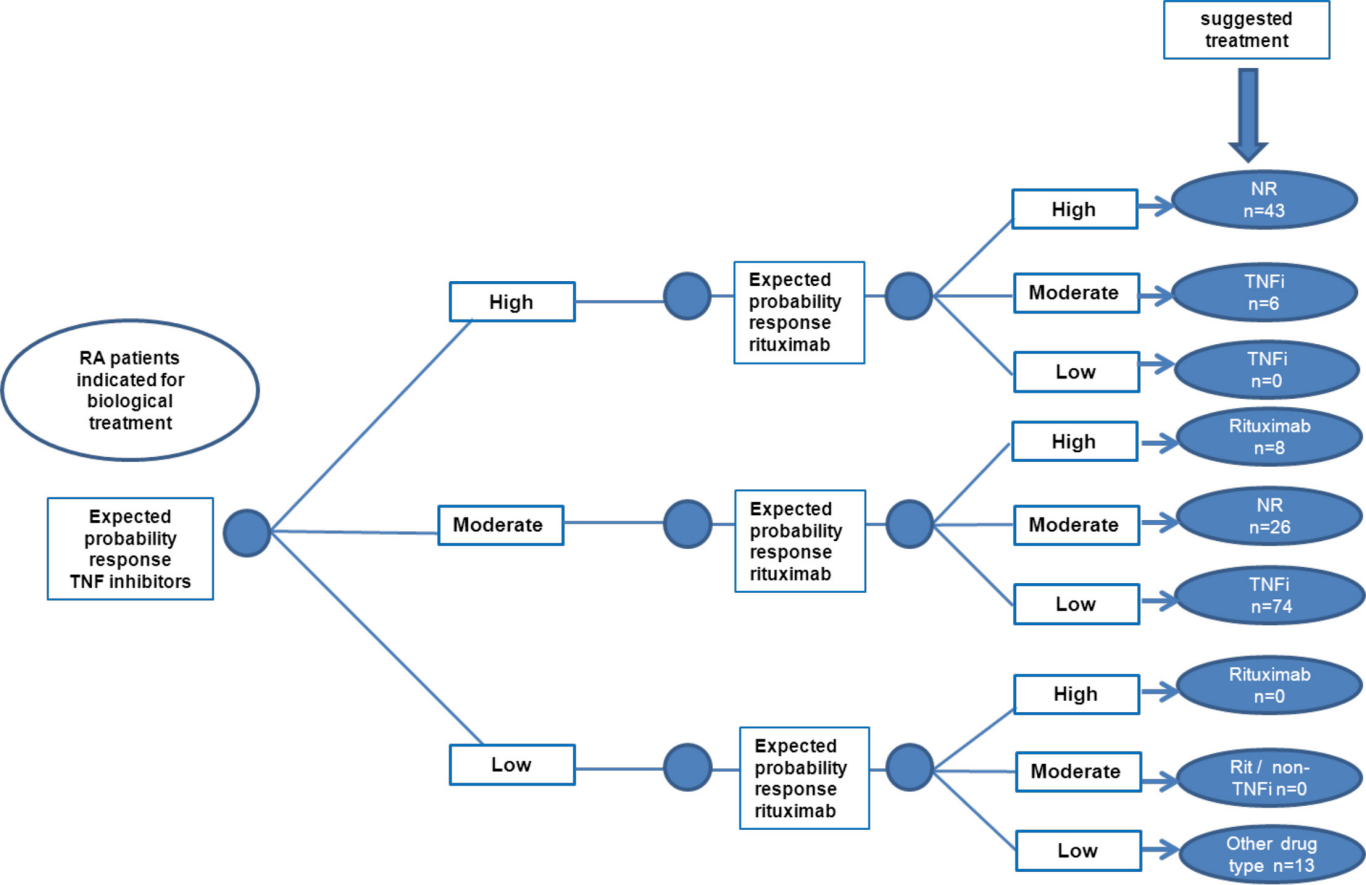
Algorithm for personalized treatment of RA patients indicated for biological treatment.

The theoretical optimal treatment choice was defined based on the expected probability (‘chance’) of response to (both) TNF-inhibitors and the expected probability of response to rituximab for an individual patient. If no distinction between drug types was present using the algorithm (i.e. probability for response to TNF-inhibitors and rituximab both ‘low’, ‘moderate’ or ‘high’), treatment decisions should be made solely by the treating physician together with the patient (‘no recommendation’). Fig 1 represents the treatment algorithm and the number of patients per specific treatment advice according to this algorithm in our patient cohort.

**Fig 2:**
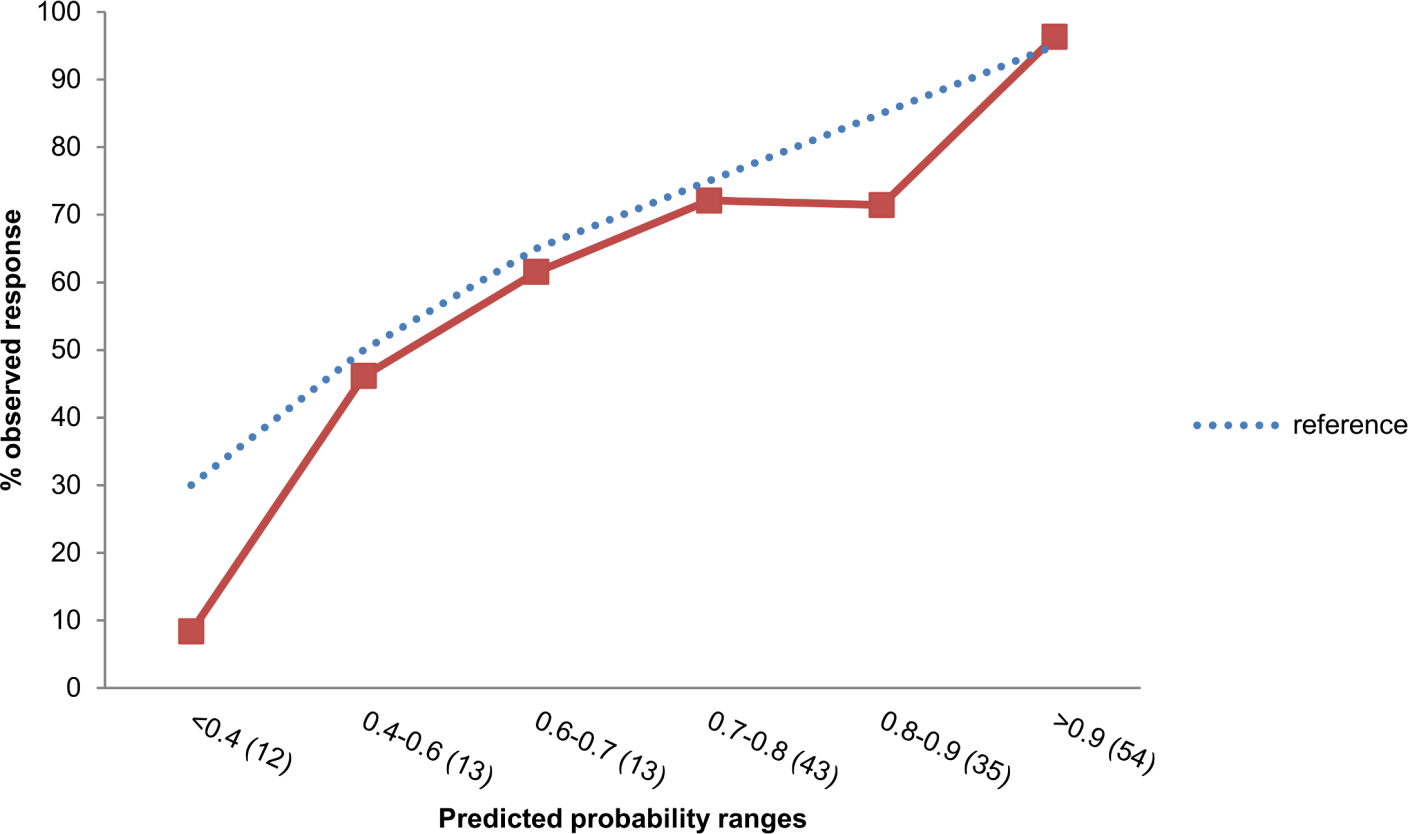
Expected probability of response versus observed probability of response.

Fig 2 shows the calibration of the bootstrapped model. In general the predicted probabilities for response seem comparable to the observed probabilities and the points are close to the line of ‘perfect fit’ indicating a fairly good prediction.

### Utility of the treatment algorithm

We compared observed responses in the cohort to predicted responses (with treatment as indicated by the algorithm) in patients where a clear treatment advice could be given (52% of the cohort; those who did not have ‘other drug type’ as recommendation or no recommendation), to assess the benefit of using this treatment algorithm in decision making. The predicted response when treated according to the algorithm was higher than observed in the cohort (around 10% beneficial; **Table 3**). The probability of response was clearly lower if the patients were treated with the other drug instead of the one indicated.

**Table 3:** Utility of the algorithm when compared to observed response.

Recommended treatment	Number of patients with recommendation in cohort	Actual treatment received	Predicted probability assuming treatment according to algorithm (mean)	Predicted probability assuming treatment contrary to algorithm (mean)	Observed probability
TNF-inhibitor	80	76TNF-inhibitor 4 Rituximab	73.2%	22.3%	63.7%
Rituximab	8	5 Rituximab 3 TNF-inhibitor	94.7%	75%	83.2%

## Discussion

The response to biological agents is heterogeneous between RA patients who have failed on DMARD treatment, [11, 20–23] and the use of these biological therapies is associated with significant risk of adverse effects and considerable costs.[24, 25] Treatment algorithms that are based in part on the use of biomarkers able to predict response to biological agents may be helpful to improve cost-effectiveness of biological treatment.[11] In this analysis, a treatment algorithm was developed for RA patients in whom biological treatment was indicated, using a prediction score based on MRP8/14 levels and clinical parameters. According to the score higher MRP8/14 levels and higher DAS28 at baseline are associated with an increased probability of response. Positivity for IgM RF and high HAQ score at baseline, as well as previous use of TNF-inhibitors are associated with a decreased probability of response.

There are numerous studies investigating biomarkers for the prediction of treatment response. Several clinical factors, such as non-smoker status, good functional ability, normal body mass index, concomitant use of DMARDs and NSAIDs and having a high disease activity at baseline, have been associated with good response to TNF-inhibitors.[26–30] The presence of IgM, RF and ACPA in relationship to response to TNF-inhibitors has been investigated in several studies with conflicting results.[31–33] In studies with rituximab, initial reports had pointed out RF positive rather than ACPA positive patients may be the best candidates to rituximab, but some studies showed that both ACPA and RF positivity is a predictor for good response.[34–36] It is currently recommended to prescribe a biological agent other than rituximab in RF and ACPA-double negative RA patients.[11, 37] Together, based on currently available data, it appears unlikely that one single factor can be used to predict response to all biological agents. Combination of multiple markers is a more promising way to improve the performance of a biomarker-guided strategy in RA patients. Since MRP8/14 is a relatively stable protein, which can easily be measured in serum by an enzyme-linked immunosorbent assay (ELISA), it is a feasible candidate as one of the predictors of response in a treatment algorithm to be used in clinical practice. Other markers in our prediction model for response, such as IgM RF, HAQ and DAS28, are also easy to measure in routine clinical practice.

The 2013 EULAR recommendations for the management of RA [2] recommend the use of TNF-inhibitors, abatacept, tocilizumab, or in some cases rituximab in RA patients who have failed conventional DMARDs, without further stratification.An initial algorithm has recently been described for patients who failed on a first TNF-inhibitor in which treatment decisions were based on whether the patient was a primary non-responder to the first TNF-inhibitor (defined as no clinical response 12–16 weeks after initiation of treatment) or a secondary non-responder (defined as initial clinical improvement followed by loss of response >24 weeks after initiation of TNF-inhibitor treatment), and based on autoantibody positivity.[11] The treatment recommendations were to switch to another TNF-inhibitor or to start a biological with another mechanism of action respectively. The treatment algorithm presented here focuses on RA patients who start biologic treatment irrespective whether they already failed a previous biological, and includes the recently identified MRP8/14 biomarker.[13, 14] We found that prediction of response using MRP8/14 levels along with clinical predictors has potential in personalizing treatment for RA patients starting biological anti-rheumatic treatment.

A clear limitation of our study is the lack of patients using biologics other than TNF-inhibitors or rituximab. Another limitation of our study is the relatively small sample size of the groups. More studies exploring the value of MRP8/14 as a predictive biomarker for treatment response will be needed to confirm the findings in independent cohorts and to extend the prediction score to the use of other biologicals. The current study provides the rationale to conduct such studies.

The prediction model could perhaps be improved when more variables are used, but we were limited to testing only eighth variables with our data (43 outcomes (i.e. non-responders)) based on recommendations for logistic regression analysis.[38]

Taken together, we have shown that MRP8/14 holds promise as a predictor for treatment response on TNF-inhibiting biologicals and rituximab and this biomarker might be a useful tool in an algorithm facilitating the decision making on which type of biological treatment has the best chance of success in individual RA patients.

**Key Messages**

1. MRP8/14 holds promise as a predictor for treatment response.
2. A treatment algorithm was developed based on the predictive value of MRP8/14 serum levels when combined with clinical parameters.
3. This biomarker might be a useful tool in an algorithm facilitating the decision making on which type of biological treatment has the best chance of success in individual RA patients.
